# The association between attendings’ feedback and residents’ reporting of near-misses

**DOI:** 10.1186/s13104-019-4395-9

**Published:** 2019-06-24

**Authors:** Sukhesh Sudan, Philip Lewalski, Judy Arnetz, John Vanschagen, Bengt Arnetz

**Affiliations:** 1 Department of Family Medicine, Michigan State University, College of Human Medicine, 15 Michigan St. NE, Grand Rapids, MI 49503 USA; 2 Department of Emergency Medicine, Wayne State University, Detroit, MI USA

**Keywords:** Residents, Feedback, Attendings, Near-misses

## Abstract

**Objective:**

Accreditation Council for Graduate Medical Education’s Clinical Learning Environment Review report suggests that residents in clinical learning environments underreport their near-misses, an indicator of patient safety. Furthermore, when the residents report these events, they receive little feedback from their attendings. Although, various studies emphasize the importance of feedback given to the residents, the association between feedback and patient safety has not been explored. This study was conducted in 28 emergency medicine residents in a level 1 trauma center. A recent study in the same cohort found that residents’ stress biomarker levels and patient acuity were positively related to their near-misses reports. The current study hypothesizes that residents that receive constructive feedback on their performance from their attendings would report more near-misses.

**Results:**

Linear regression was used to determine whether ratings of attendings’ feedback predicted residents’ reports of near-misses. Feedback was positively related to residents’ near-misses reports even after controlling for patient acuity and stress biomarker levels. This may be due to the residents becoming more aware of what a near miss is or it may also be that constructive feedback encourages them to report more near-misses as they may view this as a learning opportunity.

## Introduction

Residents in training in clinical learning environments are at increased risk of experiencing patient safety events [1, 2]. Residents’ stress, duty hours and fatigue have been linked to patient safety in clinical learning environments [1–4]. Furthermore, feedback given to the residents by their attendings has been purported to be associated with patient safety [5, 6]. Feedback on the residents’ performance is critical since it enables the residents to learn from their mistakes by discussing with the attendings as to how their performance can be improved [6].

The Clinical Learning Environment Review (CLER) program of the Accreditation Council for Graduate Medical Education (ACGME) conducts site visits every 2 years to assess teaching hospitals across the United States on six focus areas [7]. CLER identifies patient safety as one of its important focus areas and its 2018 report lists the challenges to patient safety in clinical learning environments [6]. The report suggests that residents in clinical learning environments frequently underreport their near-miss encounters due to a lack of understanding of what a near-miss is or because they are not familiar with the mechanisms in place to report such events [6]. Additionally, when the residents reported their near-misses, they received little feedback from their attendings on their performance. According to a recent CLER site visit report, there was a nominal median increase of 0.4% in feedback received by the residents from 2016 to 2018 [6]. However, the report did not explore the association between the attendings’ feedback and patient safety, specifically, whether constructive feedback by the attendings on residents’ performance increases reporting of near-misses.

Prior work from our group reported that residents’ biological stress measured by Tumor Necrosis Factor-alpha (TNF-α) was related to their reported near-misses in a large level 1 trauma center [1]. Furthermore, residents that cared for more trauma patients, a proxy for patient acuity, reported more near-misses. However, that study did not account for the effect of the attendings’ feedback on the residents’ near-misses. Based on the CLER report [6] stressing the importance of feedback to promote patient safety, the current study in the same resident cohort posits that residents that receive constructive feedback on their performance from their attendings would report more-near-misses even after accounting for patient acuity, residents’ stress levels and work-related exhaustion.

## Main text

### Participants and clinical setting

The study was conducted in the emergency department (ED) of a level 1 trauma center in 2011. There was a total of 42 emergency medicine residents of which 34 (81%) consented. Complete data was secured from 28 (67%). The participants were between 26 and 35 years of age (Mean = 29.4, SD = 2.3), primarily males (71%), and in their second (60.7%) or third year (39.3%) of residency. The residents’ program director was not involved in recruitment or data collection to avoid undue pressure on the residents to consent to participate. Rather, a senior Emergency Medicine physician who did not practice on a regular basis in the ED provided information about the study to the residents. The residents’ perceptions of the clinical environment were assessed after a rest period to control for the effect of shift-related fatigue. Over half of the residents reported having rested between 4 and 8 h prior to the start of the shift, about 38% rested over 8 h and about 7% had rested less than 4 h. The study design and reporting follow the Strengthening the Reporting of Observational Studies in Epidemiology guidelines [1].

### Data collection

The residents’ rating of their attendings’ feedback was assessed 1 week before the shift in order to capture the residents’ overall assessment of the clinical learning environment and to ensure that these ratings were not affected by shift-related factors. The study used the Quality-Work-Competence (QWC) questionnaires to assess residents’ ratings of feedback from their attendings on their performance as well as residents’ chronic exhaustion level rather than just daily fluctuations (Table 1; [8]). Responses for both QWC scales were summed to a total score and converted to a percentage, with higher scores indicating higher feedback and work-related exhaustion.

**Table 1:** Measures used in the study

Measures	Items	Response scale
Patient acuity (sum score of two items; scores range from 0 to 20 or more)	1. Number of critically-ill patients cared for during the shift 2. Number of trauma patients cared for during the shift	0 to 10 or more
QWC^a^ work-related exhaustion (sum score of three items; scores range from 0 to 100)	1. I feel emotionally drained after work 2. I feel worn out after work 3. I feel tired when I think about work	Likert-type 1 (never) to 5 (daily)
Biological biomarker	Pre-shift serum Tumor-Necrosis-Factor-alpha levels	–
QWC feedback (sum score of three items; scores range from 0 to 100)	1. Does your supervisor make it clear what is expected of you in your work 2. Does your supervisor let you know when you have done a job well 3. Does your supervisor let you know when you have done a job poorly	Likert-type 1 (not at all) to 4 (To a great extent)
Reported near-misses		
Residents’ self-reports	“Did you have any near-misses”	None, one or more than one

^a^Quality-work-competence questionnaire

Collection of serum biomarkers was conducted pre-shift, immediately prior to starting work (around 7 am). Blood samples (10 mL via venipuncture) were collected and were drawn on-site in the ED, placed on ice, and immediately transported to the laboratory. Samples were centrifuged, and plasma was stored at − 80 °C for later measurement of the biological stress biomarker TNF-α, using routine, commercially available human Enzyme Linked Immunosorbent Assay kits [1].

Immediately after the shift ended (around 4 pm), residents were asked to report how many critically-ill and trauma patients, respectively, they had cared for during the shift using an 11-point scale (from 0 to 10 or greater). These two measures were summed to denote patient acuity. The residents also rated the number of near-misses that they had experienced during the shift (Table 1). A near-miss was defined at the participating hospital as ‘any process variation that did not reach the patient, employee or visitor, but for which a recurrence carries a significant chance of a serious adverse event’.

### Institutional review

The Institutional Review Board of the university and the research office of the trauma center approved the study. All participating residents provided their written informed consent prior to participation.

### Statistical analysis

Statistical analysis was conducted using IBM SPSS statistics, V.25, 2018 (IBM Corp, Armonk, NY). Bivariate analysis using Spearman’s rho was used to examine correlations between the independent factors: patient acuity, TNF-α, work-related exhaustion, feedback and the outcome near-misses reported by the residents. Linear regression analysis was used to predict residents’ reports of near-misses. In the first step, patient acuity, TNF-α and work-related exhaustion were entered as predictors of reported near-misses. In the final step, residents’ ratings of feedback from their attendings were added. Statistical significance was set at a two-sided p value of < 0.05.

### Results

Bivariate associations and descriptive statistics are depicted in Table 2. The 28 participating residents reported a total of 8 near-misses (Mean = 0.29). Patient acuity was significantly related to residents’ reports of near-misses. Neither residents’ TNF-α levels, work-related exhaustion, nor feedback was related to reported near-misses in bivariate correlation analyses.

**Table 2:** Bivariate associations (Spearman’s rho) between patient acuity (1), biological biomarker (2), work-related exhaustion (3), feedback (4) and reported near-misses (5); n = 28

	Mean	SD	1	2	3	4	5
1 Patient acuity	3	1.92	–				
2 Tumor Necrosis Factor-alpha	52.06	184.68	0.23	–			
3 Work-related exhaustion	62.50	21.22	− 0.06	− 0.11	–		
4 Feedback	70.63	16.06	− 0.14	0.02	− 0.15	–	
5 Near-misses (resident reports)	0.29	0.54	*0.56* **	0.25	− 0.16	0.24	–

SD: standard deviation; **p < 0.01

Table 3 summarizes the results of linear regression predicting residents’ reports of near-misses. In the first step, patient acuity and TNF-α predicted residents’ reports of near-misses with the model accounting for 59% of the explained variance. When feedback was added in the second step, the explained variance increased to 69%.

**Table 3:** Predicting residents’ self-reports of near-misses using linear regression (n = 28)

	Step 1			Step 2		
	β	S.E	B	β	S.E	B
Patient acuity	*0.110*	0.042	0.397*	*0.127*	0.038	0.459**
Tumor Necrosis Factor-alpha	*0.001*	0	0.455**	*0.001*	0	0.476**
Work-related exhaustion	− 0.004	0.003	− 0.144	− 0.001	0.003	− 0.038
Feedback				*0.011*	0.004	0.337*
R^2^	0.595			0.693		

β: Unstandardized beta; S.E: standard error; B: standardized beta; *p < 0.05; **p < 0.01

### Discussion

In partial support of our hypothesis, we found a positive association between how residents rated feedback in general from their attendings, and their reported near-misses. However, the association was only significant in the multivariate analysis, when patient acuity and pre-shift TNF-α were included in the model. When feedback was added to the model, the explained variance increased by 10%. A higher number of reported near-misses when residents give higher ratings to the feedback from their attendings may be due to residents becoming more aware when he or she commits a near-miss. Alternatively, when the attendings’ provide regular and constructive feedback on the residents’ performance, this may encourage them to report any near-misses encountered as they may view this as a learning opportunity. CLER findings suggest that a clear majority of residents do not report their near-misses due to a lack of knowledge of what a near-miss is [6]. It also states that attendings in clinical learning environments give little feedback to the residents on their performance. In addition, when the feedback is given, there is little to no discussion between the residents and their attendings on how their performance can be improved. However, the CLER report does not explore the association between attendings’ feedback to the residents and patient safety.

The impact of patient acuity and TNF-α on residents’ reporting of near-misses in this same population has been previously reported [1]. Residents working in EDs need to make time-sensitive decisions in a high stress environment and are thus at increased risk to experience adverse events or near-misses [1, 2]. The complexity of the ED patients further contributes to a higher patient acuity, which may have implications for patient safety. However, this study found that even after controlling for patient acuity, TNF-α and exhaustion, attendings’ feedback on the residents’ performance was related to their reported near-misses.

There is a clear need to identify reliable measures of residents’ performance that allow an open and data-informed discussion between the residents and the attendings on near-misses and actual adverse medical events. Although this was a small study, the 28 participating residents reported 8 near-misses over a short time period, which is not an insignificant number. Near-misses are encountered more frequently compared to adverse patient outcomes and provide an opportunity to improve patient safety and prevent actual adverse events by having an open dialogue between the residents and the attendings since the patient was not hurt in this instance [9, 10]. Furthermore, the learning points from the near-misses can be used to devise strategies to decrease future risks when facing similar patient challenges [9, 10]. Other industries such as aviation and the nuclear power industry frequently use near-misses as a mechanism for learning and preventing future adverse events [11]. ACGME’s CLER report emphasizes that with increased focus on patient safety, the use of near-misses along with documented adverse events is warranted [6].

The current study provides a self-report measure that can be used to quantitatively assess residents’ appraisals of their attendings’ feedback on the residents’ performance. The establishment of quantitative and psychometrically validated measures are critical to be able to define and achieve high-performance clinical learning environments as well as benchmarking and learning across academic healthcare centers. This study assessed the attendings’ feedback on residents’ performance in close proximity to the actual ED shift. This allowed us to study short-term temporal relationships between the residents’ rating of the feedback they receive from attendings and reported near-misses.

### Conclusion

This pilot study suggests that the management’s efforts to encourage reporting of near-misses by residents should focus on improving the malleable factor in clinical learning environments i.e. feedback given to the residents by their attendings. Future large-scale studies should explore the association between attendings’ feedback and residents’ near-misses in different clinical learning environments to validate the findings from this study.

## Limitations

The sample size was small and included residents from only one emergency department. Furthermore, actual numbers of documented adverse medical events were not assessed.

## Data Availability

The data sets used and/or analyzed during the current study are available from the corresponding author on reasonable request.

## Abbreviations

CLER: Clinical Learning Environment Review
ACGME: Accreditation Council for Graduate Medical Education
TNF-α: Tumor Necrosis Factor-alpha
ED: emergency department
QWC: Quality-Work-Competence
